# Patient Perspectives on the “Future Patient” Telerehabilitation Program for Atrial Fibrillation: Qualitative Study

**DOI:** 10.2196/68663

**Published:** 2025-08-19

**Authors:** Elisabet Dortea Ragnvaldsdóttir Joensen, Andi Eie Albertsen, Helle Spindler, Katja Møller Jensen, Lars Frost, Lars Dittmann, Mathushan Gunasegaram, Søren Paaske Johnsen, Mads Rovsing Jochumsen, Dorthe Svenstrup, Birthe Dinesen

**Affiliations:** 1 Department of Health Science and Technology, Aalborg University, Selma Lagerløfsvej 249, Aalborg, 9260, Denmark, 45 20771373; 2 Regionshospitalet Viborg, Viborg, Denmark; 3 Department of Psychology and Behavioural Sciences, Aarhus University, Aarhus, Denmark; 4 Regionshospitalet Silkeborg, Silkeborg, Denmark; 5 Department of Electrical and Photonics Engineering, Danish Technical University, Copenhagen, Denmark; 6 Danish Center for Health Services Research, Aalborg University, Aalborg, Denmark

**Keywords:** atrial fibrillation, telerehabilitation, qualitative interviews, patient education, home monitoring

## Abstract

**Background:**

Atrial fibrillation (AF) is a prevalent chronic condition with increasing incidence worldwide. AF increases the risks of stroke, heart failure, and myocardial infarction and imposes a substantial burden on the health care system. Cardiac rehabilitation programs, while effective, often have low patient adherence. Recent evidence suggests that cardiac telerehabilitation, where patients are given home monitoring devices, could enhance adherence and outcomes. The program “Future Patient—Telerehabilitation of Patients with AF” (FP-AF) was created to assess the effects and potential benefits of cardiac telerehabilitation on patients with AF.

**Objective:**

The objective of this study is to explore the experiences of patients participating in the FP-AF program.

**Methods:**

This qualitative sub-study is part of the multicenter, randomized controlled FP-AF trial, which included 208 patients. Semi-structured interviews were conducted on 14 patients, randomly selected from participants in the intervention arm of the FP-AF program. The patient interviews, guided by self-determination theory, focused on patients’ experiences with the FP-AF program, including the use of telerehabilitation technologies and a web-based portal called the “HeartPortal.” Interview responses were analyzed using NVivo software (version 14.0; QSR International), with thematic coding based on interview guides and methodological guidance elaborated by Brinkmann & Kvale. The study adhered to ethical guidelines, with informed consent obtained from all participants.

**Results:**

Based on the interviews, the following themes were identified: the home monitoring devices are viewed positively by the patients; the HeartPortal is a useful digital toolbox; patients develop new coping strategies for living with AF; the measured values are useful for the patients; the community of practice is beneficial; and the FP-AF program creates a sense of security.

**Conclusions:**

Participation in the FP-AF program enhanced patients’ sense of security, empowerment, and knowledge about AF. This improvement was due largely to a combination of patients’ use of the HeartPortal and the educational sessions at health care centers. Telerehabilitation for patients with AF may be a useful way of researching this group of patients with a focus on rehabilitation and may be an effective means of offering rehabilitation to this group in the future.

## Introduction

Atrial fibrillation (AF) is a chronic cardiovascular condition with a lifetime risk affecting about 1 out of 3-5 individuals aged 45 years or older, depending on their risk factor profile [1-3]. Risk factors for AF include weight, hypertension, physical activity, diet, alcohol consumption, and smoking status, as well as comorbidities such as type 2 diabetes mellitus, sleep apnea, heart failure, and myocardial infarction [1,3]. The incidence of AF is growing due to an aging population, improved opportunistic screening for asymptomatic AF, and an increase in modifiable risk factors [1,2,4]. Untreated AF is associated with significant risks, including a fivefold increase in the risk of stroke and heart failure, as well as a twofold increase in the risk of myocardial infarction and excess mortality [1,5]. In addition, AF imposes a substantial economic burden on health care systems [6].

AF is known to negatively affect quality of life (QoL) and restrict patients’ ability to carry out daily activities [7]. However, these negative impacts may be mitigated through patient empowerment [8]. The World Health Organization defines empowerment as “a process through which people gain greater control over decisions and actions affecting their health” [9]. Health care professionals can promote patient empowerment by implementing a patient-centered approach [10,11], with a key focus on enhancing patients’ understanding of their condition [11]. Patients with AF may benefit from cardiac rehabilitation (CR) programs specifically tailored to help them manage and live with their condition [12]. A Cochrane review highlights that exercise-based rehabilitation for patients with AF reduces symptoms and recurrence and improves QoL and exercise capacity [13].

CR includes health management interventions that provide patients with the necessary knowledge and support to manage their disease through patient education, exercise, risk-management strategies, and psychological support [12]. A systematic review of educational interventions for patients with AF states that patient education is associated with a decrease in mortality and readmission, as well as having a positive impact on psychological factors such as anxiety, depression, and QoL [14]. Despite these benefits, studies have shown that adherence to CR is low. Factors associated with lower adherence to CR include female gender, older age, unemployment, comorbidities, and geographical barriers [15,16].

A Cochrane review [17] comparing home-based and center-based CR found that both types of CR were similar in their effects on QoL, modifiable risk factors, exercise capacity, mortality, and hospital admission. The Cochrane review found a small but significantly higher completion rate for home-based CR compared to the center-based CR [17].

A recent literature review by Owen and O’Carroll [18] found that cardiac telerehabilitation (CTR) had a level of effectiveness equal to that of center-based CR in outcomes such as physical activity, weight, blood pressure, QoL, depression, and anxiety. Additionally, the telerehabilitation groups’ adherence to the rehabilitation program was higher than center-based rehabilitation [18]. In another study, Cai et al [19] found that patients with AF undergoing CTR had significantly increased cardiac capacity compared to those in conventional CR. Similar findings are reported by Pagliari et al [20], who found that CTR led to significantly increased exercise capacity. Furthermore, Cai et al [19] found significant improvements in health beliefs and physical activity in both groups. These findings indicate that CTR, which takes place in the patient’s home environment, could be a suitable alternative to CR because it would generate a potentially higher level of adherence to rehabilitation programs. CTR may either supplement or serve as an alternative to center-based CR. A CTR solution delivers one or more rehabilitation modules directly to the patient through technologies such as wearables, smartphones, and video calls, allowing patients to participate from their own homes [21]. In order to assess the effects of CTR on patients with AF, the “Future Patient” program has been developed.

The educational CTR program “Future Patient—Telerehabilitation of Patients With AF” was developed through a co-creation process involving patients with AF, their relatives, and researchers. The program was evaluated in a pilot study by Dinesen et al [22], where it was found to be useful by patients with AF and their relatives. In particular, our CTR program was found to enhance patients’ sense of security, increase their knowledge about symptom management, and promote a community of practice that connected patients and their relatives with health care professionals. Following the pilot study, the FP-AF program is now being evaluated in a multicenter, mixed-methods, randomized controlled trial, which began enrolling patients in January 2023 and is expected to conclude in June 2025.

This study aimed to explore the experiences of patients with AF participating in the FP-AF program.

## Methods

### Qualitative Study

The present study is a qualitative sub-study within the multicenter, mixed-methods randomized controlled trial on the FP-AF program (FP-AF study), which includes a total of 208 patients [23]. This qualitative sub-study uses a triangulation of data collection techniques: document analysis, patient observation, and semi-structured interviews with patients from the intervention group. Furthermore, a user panel was established, with meetings held twice a year during the FP-AF study. This study was reported in accordance with the Consolidated Criteria for Reporting Qualitative Research checklist [24].

### Participants

Patients diagnosed with AF at the Departments of Cardiology at Silkeborg, Viborg, and Skive Regional Hospitals (Denmark) were assessed for eligibility for the FP-AF study. The inclusion criteria were as follows: the patient must be diagnosed with AF; be an adult aged 18 years or older; live in the Skive, Viborg, or Silkeborg municipalities; live at home and be capable of caring for themselves; and possess basic computer skills or have a relative or friend with basic computer skills. The exclusion criteria were as follows: pregnancy; refusal or inability to cooperate; patients who did not speak, read, or understand Danish; and patients with a life expectancy of less than a year, based on clinical judgment and underlying medical conditions.

In selecting the interviewed patients, we used a random selection process, ensured equal distribution of females and males, and selected those patients who had participated in the patient education module at the health care centers. The participants were contacted by telephone by a research assistant and invited to participate in the interviews. In total, 18 randomly selected patients were contacted, of whom 3 were unable to participate on the suggested dates and 1 did not wish to participate. A total of 14 patients were interviewed. The patients have not recieved any compensation for their participation in the interviews. 

### Ethical Considerations

The FP-AF study was conducted in accordance with the Helsinki Declaration, and all participants were asked to sign an informed consent form before entering the study. Upon enrollment in the FP-AF study, all participants agreed to participate in interviews regarding their experiences with the study. The FP-AF study has been approved by the regional Ethics Committee (N-20220056) and is listed in ClinicalTrials.gov (NCT06101485). Participants were informed that they have the right to withdraw their consent at any time during the study, and the reason for their withdrawal will also be documented if participants so wish. An agreement on data sharing has been established between the participants and the researchers.

### Interventions

**Figure 1.**
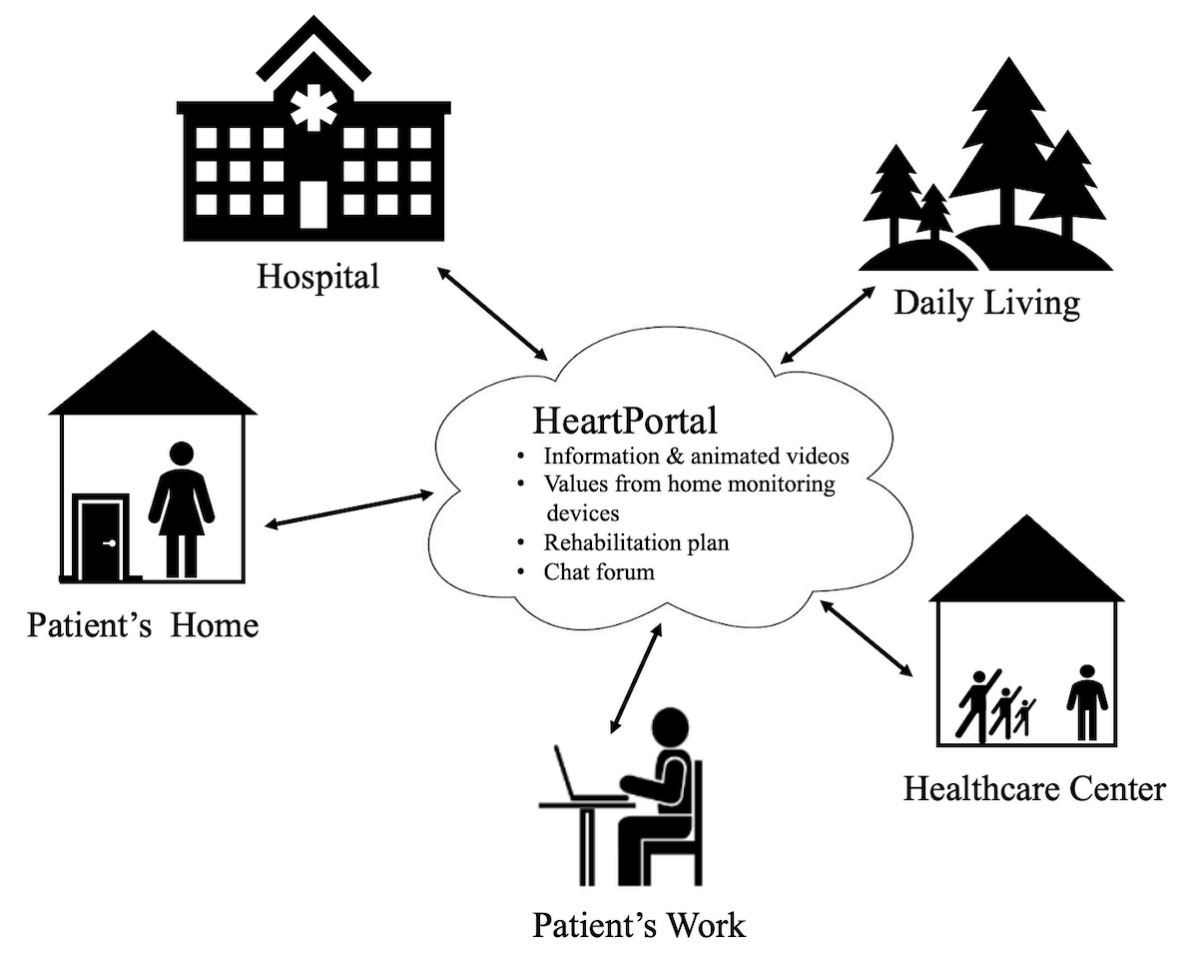
Context of the FP-AF program. FP-AF: Future Patient—Telerehabilitation of Patients with AF.

The FP-AF program is targeted at both patients with AF and their relatives. The program consists of two modules: (1) an education and monitoring module using telerehabilitation technologies, lasting 4 months and (2) a follow-up module, where the patients use their own personal devices to measure steps and have access to the HeartPortal, lasting 3 months. Upon enrollment, patients in the intervention group were given several home monitoring devices, including a blood pressure monitor, weight scale, activity tracker, electrocardiogram (ECG) monitor, and sleep sensor. All patients had individual meetings with the project nurses, during which they received instruction on how to use the technologies. The HeartPortal is a web-based portal accessible to patients and health care professionals. Project nurses review the patients’ measurements twice a week and have continuing contact with the patients through the HeartPortal. Patients could give their consent to allow their relatives to be able to access the HeartPortal. In addition, patients and their relatives were invited to participate in an educational course at the local health care center, covering themes such as living with AF, recognizing symptoms of AF, managing the disease using digital technologies, and supporting a spouse or partner with AF. Patients in the control group received the conventional AF education delivered in person at the hospital. The education program consists of 4 sessions, each lasting 3 hours. The context of the FP-AF program can be seen in Figure 1.

### The HeartPortal

The HeartPortal was developed through a participatory design process and functions as both a digital toolbox and a web-based learning module. Patients and their relatives in the intervention group have access to the HeartPortal, where they can receive information about AF through text and animated short films. They can also view visualizations of their measured data (blood pressure, weight, steps, and sleep) and communicate via chat and video with health care professionals at the hospital and health care centers. The data from the ECG can be accessed on a separate platform.

### Theoretical Approach: Self-Determination Theory

The FP-AF program is based on self-determination theory (SDT). SDT highlights motivation as an essential component of any successful rehabilitation [25]. In this light, SDT identifies 3 basic needs for human motivation: (1) autonomy, meaning that the patient identifies with the goals of rehabilitation and values these goals as personally important; (2) competence, meaning that patients believe themselves to have (acquired) the necessary skills and knowledge to achieve their goals and receive appropriate feedback and guidance; and (3) relatedness, meaning that the health care professionals and social network create an environment where the patient feels supported, respected, and understood. To ensure continuous engagement, the motivation must be intrinsic to the patient, such that all 3 primary needs must be supported simultaneously [25].

### Document Analysis and Patient Observation

Document analysis was conducted through a systematic evaluation of written materials, including homepages on digital health strategies, rehabilitation policies, and homepages from the involved health care organizations; these materials enabled us to assemble relevant background knowledge for the context of this study. The patient observation was conducted during the education modules held at the health care centers, and the aim here was to observe the quality and level of patient engagement.

### Semi-Structured Interviews

Semi-structured exploratory interviews, inspired by Brinkmann and Kvale [26], were conducted with 14 patients from the intervention group in the FP-AF study. These interviews took place upon the completion of the program. An interview guide was designed in several steps: (1) Background questions were formulated in order to start the interview, eg, the patient is asked to present themselves and their disease; (2) based on the theoretical framework, key themes were extracted for the interview guide, eg, motivation, competences, and autonomy; (3) a total of 4 interviews were transcribed, read, and coded by 2 researchers individually. After the coding, the researchers compared the codes to ensure that they shared the same understanding of the codes and to ensure intersubjectivity. Via this process, new themes and questions were identified for the interview guide, eg, on the importance of participation in education at the local health care center; and (4) finally, the interview guide was pilot tested, adjusted, and prepared for use. The interview guide covered the following themes: the use of technologies and the HeartPortal, the patients’ experiences with the telerehabilitation program, their AF knowledge, competencies, communication with health care professionals, and the perceived advantages or disadvantages of the program.

The interviews were conducted by the first (EDRJ, BSc, student assistant) and last (BD, PhD, professor) female authors, who have years of experience conducting qualitative research. The interviewers had no prior personal relationships with the patients. The interviews took place in the patients’ homes and lasted 30‐60 minutes. All interviews were tape-recorded, and the interviewers took notes throughout. Condensed data were presented for a user panel in the FP-AF program. After 10 interviews, the research team agreed that they had reached a point of data saturation, defined as the point where no new themes or information emerged. The aim of the user panel has been to have a panel of patients and their relatives who can give second opinions and feedback on relevant issues in the project. The panel consisted of 9 patients who had participated in the FP-AF program, with new patients joining the panel as the study progressed. Preliminary findings from the interviews were discussed at the user panel meeting in May 2024 in order to validate whether data saturation had been reached. After the discussion, the patients advised the research team to conduct more interviews. After 4 more interviews, the research team found that no new themes or information emerged and concluded that data saturation was reached.

### Analysis of Data

A research assistant transcribed all interviews into text files using Microsoft Word. Any identifying information regarding the patients was removed from the transcript, and their names were anonymized with alias names. The transcribed interviews were then coded in NVivo (version 14.0; QSR International), guided by the SDT theoretical framework and by interview analysis methodologies developed by Brinkmann and Kvale [26]. Two researchers developed the code tree, defining overall themes and subthemes based on the interview guide, concepts derived from the theoretical framework, and key findings from the analysis of 3 random interviews. Before the analysis of the interviews, the code tree was reviewed and discussed by the 2 researchers to ensure intersubjectivity. The code tree was then used for the analysis of the interviews with a focus on the experiences of patients with AF participating in the FP-AF study. The data have been condensed and are presented in Table 1.

**Table 1.** Identified themes and subthemes from interviewed patients in the FP-AF^a^ study.

Themes	Subthemes
Devices have many functions	User-friendly technology (n=11) ECG^b^ creates a sense of security (n=3) Step counter motivates me to exercise (n=5)
HeartPortal as a digital toolbox	Functions like a toolbox to navigate AF (n=5) Used as a communication platform with health care professionals (n=8) Have not used the HeartPortal frequently (n=5)
Coping strategies for living with AF	Increased knowledge to handle own symptoms (n=12) Feel empowered to handle own disease (n=4)
Measured values are useful	Give an overview of own data (n=5) Creates a sense of security (n=6) Careful not to be overly concerned with measuring my values (n=2)
Community of practice	Community of practice with peers is beneficial (n=10) Experienced patients with AF already have the knowledge (n=2) Meeting and talking to other patients can increase your awareness negatively (n=1)
FP-AF program creates a sense of security	Information provided by the health care center is useful (n=10) The patient education creates a sense of security (n=11) From a sense of unease to a sense of calm (n=2) Being monitored at home is beneficial (n=2) No need for education at health care center (n=2)

a FP-AF: Future Patient—Telerehabilitation of Patients with Atrial Fibrillation.

b ECG: electrocardiogram.

## Results

The interviewed patients ranged in age from 58 to 93 (mean 70 [SD 8] years). There was an equal distribution of men and women in the interviews, and the patients were predominantly diagnosed with paroxysmal AF (n=11, 79%), married (n=10, 71%), had a vocational qualification (n=9, 64%), and were largely retired (n=10, 71%). Table 2 shows the baseline characteristics of the patients.

**Table 2.** Baseline characteristics of patients interviewed (N=14) in the FP-AF^a^ study.

Variable	Value
Gender, n (%)	
Males	7 (50)
Females	7 (50)
Age (years), mean (SD)	
Males	70.4 (11.0)
Females	70 (5.3)
Primary diagnosis, n (%)	
Paroxysmal	11 (78.6)
Persistent	2 (14.3)
Permanent	1 (7.1)
Clinical parameters, mean (SD)	
Weight (kg)	82 (16.5)
Height (cm)	171.6 (9.7)
Systolic blood pressure (mmHg)	127.6 (12.5)
Diastolic blood pressure (mmHg)	84.1 (8.3)
Pulse (beats/min)	62.6 (10.4)
Ejection fraction (%)	58.2 (6.7)
CHA_2_DS_2_VASc score	1.9 (1.4)
Secondary diagnosis, n (%)	
Heart failure	1 (7.1)
Hypertension	6 (42.9)
Peripheral arterial disease	1 (7.1)
Aortic plaques	1 (7.1)
No secondary diagnosis	5 (35.8)
Civil status, n (%)	
Single	1 (7.1)
Married or living with a partner	10 (71.4)
Widow or widower	3 (21.4)
Education, n (%)	
Unskilled worker	3 (21.4)
Skilled worker	7 (50)
Master’s degree	4 (28.6)
Work status, n (%)	
Works under 20 hours/week	1 (7.1)
Works 20‐36 hours/week	1 (7.1)
Works full time 37 hours/week	2 (14.3)
Retired	10 (71.4)

a FP-AF: Future Patient—Telerehabilitation of Patients with Atrial Fibrillation.

b CHA_2_DS_2_VASc score: used to assess the risk of stroke in individuals with atrial fibrillation (C: congestive heart failues, H: hypertension, A: age 75 or above (2 points), D: diabetes mellitus, S: stroke/TIA/thromboemolism (2 points), V: vascular disease, A: age 65-74 years, Sc: sex category female).

### Findings

In total, 7 overall themes were generated: “Devices have many functions,” “HeartPortal as a digital toolbox,” “Coping strategies living with AF,” “Measured values are useful,” “Community of practice,” and “FP-AF program creates a sense of security.” Each theme generated several subthemes, which include a representative patient quote identified with an ID number. Table 1 gives an overview of the overall themes and subthemes generated from the interviews.

### Devices Have Many Functions for the Patients

The theme “devices have many functions” explores the patients’ experiences with using the various monitoring instruments, which include a blood pressure monitor, weight scale, activity tracker, ECG monitor, and sleep sensor. Overall, patients stated that the devices were user-friendly and easy to set up:

I found using the technologies easy; it did not bother me at all. It was easy to use, and did not require great knowledge.

[ID580]

The device mentioned most frequently by the patients was the ECG monitor, as the patients found it to create a sense of security:

I missed it when they took it away from me. Being able to measure my heart rhythm was very reassuring.

[ID580]

I can imagine that most people, including me, find the ECG important. It was the device I was most pleased about.

[ID666]

The patients also found that the devices motivated them to exercise more. This was particularly true of the step counter:

I keep track of my steps during the day. It has become a goal to walk 10,000 steps every day.

[ID726]

These statements indicate that the devices were well-received by patients for their ease of use and functionality. The ECG monitor, in particular, provided reassurance and was valued among the patients. In addition, the activity tracker motivated patients to increase their physical activity, suggesting that these devices can positively impact health behaviors and enhance patient engagement.

### The HeartPortal as a Digital Toolbox

The theme “HeartPortal as a digital toolbox” explores the patients’ experiences using the HeartPortal. Several patients found that the HeartPortal functioned like a toolbox for navigating their AF:

The HeartPortal has functioned like a toolbox, I used it in connection with the preliminary consultation I had before my ablation, where I watched the videos on the portal.

[ID789]

It’s nice to have everything gathered in one place. I have been in dialogue with the project nurse a few times, and I used it to keep track of my measurements.

[ID684]

In addition to serving as a toolbox for managing AF, patients found the HeartPortal to be a useful communication platform for communicating with health care professionals. Communication through the HeartPortal was viewed as more time-efficient, allowing patients to ask questions directly without needing to communicate through multiple intermediaries when an immediate answer was not required:

I have been in contact with the project nurse through the HeartPortal… We don’t have to call and steal each other’s time, so I think it’s great for those questions where you don’t need an answer right away.

[ID674]

The platform functions like a direct line where you can skip your doctor and a secretary at the hospital. With medicine and such, I believe that it has helped to make me calmer.

[ID623]

Nevertheless, some patients did not find that they had used the HeartPortal frequently. Some stated that they had not experienced AF during the project and therefore did not feel a significant need for the platform:

I have not used the HeartPortal so much during the project. I have written a couple of messages to the project nurse, but I have not had AF during this period, so I have not really felt the need for it.

[ID666]

Others mentioned that they were not accustomed to electronic data processing and therefore had not used the HeartPortal:

I have not really used the HeartPortal. I am not used to EDP. I could have done more, but I may not have had the biggest need.

[ID674]

The patients’ use of the HeartPortal indicates that the platform is a valuable tool for many patients, as it helps them manage their AF and facilitates easier communication with health care professionals. The communication part of the platform is especially appreciated for non-urgent communication with health care professionals. However, the usage of the HeartPortal varies among the patients, with some patients not feeling the need for it during the period of the study and others being less comfortable using digital communications due to a lack of digital literacy. This suggests that even though the HeartPortal is beneficial for patients, its use may be enhanced by addressing the needs of the individual patient regarding their confidence in the use of digital tools.

### Coping Strategies Living With AF

The theme “Coping strategies living with AF” explores the FP-AF program’s effect on patients’ coping strategies. Nearly all the interviewed patients reported gaining greater knowledge of how to manage their AF symptoms. One patient stated that the program created a curiosity about her AF:

I have learned a lot, and I have gained a greater knowledge of my illness since using the technologies… it has created a curiosity in relation to my illness, in which I’ve obtained a better understanding.

[ID674]

In addition, some patients stated that their participation in the FP-AF program made them feel more empowered to manage their AF:

I feel like I’m capable of handling my AF. Participating in the project has given me a sense of calm.

[ID580]

These findings indicate that the FP-AF program has had a positive impact on patients’ abilities to cope with their AF, as it has provided them with increased knowledge of their symptoms and created a sense of security, if not empowerment.

### Measured Values Are Useful

The theme “Measured values are useful” explores patients’ use and attitudes toward the data overview provided by their measured values. Patients felt that the measured values created a clear overview of their condition, with one patient noting that this overview even helped her lose weight during the program:

I looked a lot at the graphs of my blood pressure and weight, and so on. I actually lost a lot of weight during that time.

[ID580]

Furthermore, the patients also found that having an overview of their data created a sense of security:

What I’m doing now, measuring all these values, helps me… I immediately feel better, it has created a sense of security.

[ID684]

I couldn’t sleep before I got the ECG measure. It created a sense of security being able to see how everything was going.

[ID580]

Of the 14 patients, 2 expressed concern regarding measuring their data. They did not want to become anxious about measuring their values. One patient stated that measuring your values could make you sick:

I would not be measuring my values if not for this project. I do not think you should seek out illness, it can make you sick.

[ID686]

These perspectives indicate that while many patients find value in the data overview provided by their measured values, and that this may lead to positive outcomes such as weight loss and increased security, there were also concerns about the potential for increased anxiety and over-monitoring.

### Community of Practice

The theme “Community of practice” explores patients’ experiences in the FP-AF program, with a focus on the educational benefits of engaging with peers who also have AF. Many patients found it particularly helpful to learn from and share experiences with others in a similar situation. This community-based learning helped patients gain a greater knowledge of AF and how it affects individuals differently.

The dialogue with other patients is a big part of the education at the healthcare centres. You learn a lot by talking to others about how they’re feeling and how it affects them.

[ID742]

I got so much smarter from being with other people with the same illness. I feel it in my way, but others feel it in a completely different way, something you don’t know when you’re all alone.

[ID766]

For patients with AF who were already well-informed and not severely impacted by their AF daily, the program’s educational aspects were less useful. Some felt they did not gain new knowledge, as they were already comfortable managing their AF:

The project has not contributed anything new for me, as I am very oriented about it. I have had AF for many years and acquired a lot of knowledge.

[ID703]

Another patient stated that she felt secure in managing her AF from the beginning and thus did not see the need for the educational program:

I felt safe in my illness right away and didn’t feel like reading any more about it.

[ID623]

For this patient, participating in the program would only lead to her focusing on issues that were not at all problematic:

You can end up focusing on things that might not actually be an issue.

[ID623]

These findings indicate that the community of practice proved beneficial for most patients in enhancing their understanding of AF. Those patients with AF who were more confident in managing their disease may have felt that the program was unnecessary for them compared to others who are less familiar with their condition. Furthermore, there is a concern that participation in the program could lead to unnecessary focus on potential issues of little relevance to the patient. The differences among patients with AF indicate a need for tailored interventions that can incorporate the varying levels of their pre-existing knowledge and experience.

### FP-AF Program Creates a Sense of Security

The theme “FP-AF program creates a sense of security” explores the overall experiences of patients participating in the program. Most patients found the education at the health care center beneficial:

I am very satisfied and feel that I have received everything and more during the 4 days of instruction. I don’t think there’s anything missing or lacking in the course. Not in relation to the teaching’.

[ID789]

Patients also reported that the lectures at the health care center significantly contributed to their sense of security:

The sense of security that it has given me is worth its weight in gold.

[ID666]

A few patients noted that the project had given them a sense of calm regarding their AF:

At the beginning of my AF, I was in a dark place, and I was scared to walk alone. Today I can walk alone, and I walk 10 kilometres every second day. I am well now… being in the project gave me peace.

[ID580]

Some patients also stated that home monitoring was beneficial because it reduced the need for hospital checkups:

You can be monitored at home and don’t need to go to the hospital for check-ups so often. This is especially good for elderly people, who may not have a convenient way to transport themselves.

[ID635]

Not all the patients saw the need for the education at the health care center as necessary, however.:

I don’t take part in the patient education in the healthcare centre. For me, I don’t find it necessary to talk about my illness once a week. I’ve come to terms with it being the way it is.

[ID726]

These perspectives indicate that for most patients, the FP-AF program provides benefits in terms of education, security, and community support. The lectures at the health care center and interactions with other patients help the patients create a sense of security and understanding about their condition. However, the program’s value varies among patients, with some not feeling the need for weekly discussions or community engagement. Home monitoring is also found beneficial, particularly for those who find it difficult to travel to the hospital.

## Discussion

### Principal Findings

This study has explored the experiences of patients with AF in participating in the FP-AF program intervention arm. For most patients, the FP-AF program created an enhanced sense of security and empowerment and improved their knowledge of AF. The lectures at the health care center added to their knowledge and sense of security, while the community of practice with peers increased the patients’ understanding of the individuality of AF. Patients found the technology user-friendly, and the HeartPortal’s data overview further increased their sense of security and motivated them to additional exercise. However, the interviews also revealed that some patients felt that they did not benefit from the education at the health care centers.

These findings are consistent with existing literature that highlights the importance of a community of practice and interaction with health care professionals in the rehabilitation process [27-30]. Kenny et al [27] highlighted the important role of a support network, particularly from staff, and noted patients’ dissatisfaction with limited interaction opportunities with peers. Similarly, Anttila et al [28] emphasized that connecting with others is a crucial component of the rehabilitation experience. Furthermore, Lunde et al [29] found that a supportive individual behind a smartphone app is vital for promoting healthy behaviors following rehabilitation.

The study also revealed that the devices used in the FP-AF program were well-received and appreciated for their user-friendliness and functionality. The devices provided patients with a sense of security and motivated them to exercise, indicating a positive impact on health behaviors and patient engagement. These results align with Kenny et al’s [27] results, which showed that self-monitoring tools such as heart rate monitors, blood pressure monitors, and activity trackers improved patients’ insights into their physical condition and allowed them to track their progress, thereby enhancing their psychological well-being. This is further supported by Olofsson et al [31], who found that self-monitoring enhanced patients’ understanding of their symptoms and contributed to a higher level of autonomy [31]. In addition, patients reported that the FP-AF program made them feel empowered to deal with their AF through increased knowledge of their illness, a finding that aligns with Kenny et al’s study, which found that CTR empowered patients and resulted in greater knowledge and involvement in their recovery process [27]. Similarly, Su et al [32] found that CTR enhances patient knowledge and empowerment by equipping them with skills to modify their behavior and address everyday challenges.

The HeartPortal was identified as a valuable tool for many patients, serving as a tool for the management of AF and as a platform for easier communication with health care professionals. This finding supports Kenny et al’s [27] observation that patients value the ability to contact health care professionals as needed, highlighting the importance of both digital tools and in-person support.

The FP-AF rehabilitation program was based on SDT, where motivation is seen as a key component in any successful rehabilitation [25]. The findings in this qualitative sub-study highlight how the FP-AF rehabilitation program appears to fulfill the needs for autonomy, competence, and relatedness [25]. The program allowed patients to take control of their health, provided patient education and a community of practice, and gave patients useful tools and knowledge to manage their AF. However, a few of the interviewed patients expressed concerns about the self-monitoring aspect of the program, noting that it could lead to excessive focus on issues that may not be relevant, potentially increasing symptoms of anxiety. The relationship between self-monitoring and anxiety is complex, with heterogeneous findings across studies. For instance, Rosman et al [33] found that a self-management program incorporating self-monitoring reduced symptoms of anxiety among patients with AF. Research in other populations, however, such as those with diabetes and heart failure, found no effect of self-monitoring on psychological factors [34,35].

In summary, the study highlights the need for a balanced approach in CR programs that combines digital tools and home monitoring with in-person support and community interactions. A comprehensive rehabilitation program for patients with AF should effectively combine these digital and interpersonal elements to enhance the overall patient experience and outcomes.

The present study also revealed that some patients did not benefit from the FP-AF program. This aligns with Vonk et al’s [36] findings that some patients did not participate in CR because they did not feel the need for additional supervision or see benefit in the program’s trajectory. Furthermore, Vonk et al [36] found that some participants were reluctant to participate in the group meetings connected to CR, as they did not want to hear about other people’s problems. In the present study, some patients were less comfortable with the use of these digital technologies due to limited technical literacy, which may have affected their ability to participate in the FP-AF program. Research suggests that the degree of digital literacy may influence the likelihood of patients using telehealth technologies [37-39]. Moreover, factors such as age, socioeconomic status, race, and eHealth literacy have been found to influence patients’ engagement with these technologies [38,40]. Hesitancy to use telehealth technologies may be rooted in several factors, including difficulties operating the technology, lack of access, poor quality of telehealth appointments, and a preference for in-person care [41]. These insights suggest that while the FP-AF program offers valuable benefits for many patients, its effectiveness may vary based on the individual patient’s pre-existing knowledge and experience in managing their condition. Future telerehabilitation for patients with AF needs to tailor interventions to better address the needs of the patients at different stages of managing their condition.

### Limitations

The interviewed participants were recruited with the criteria of having participated in the patient education at the health care centers, which may introduce selection bias. We evaluated patients over a short period of time, and since AF is a chronic condition, a longer follow-up period could have been beneficial for exploring the long-term effects of telerehabilitation. Furthermore, it should be noted that the majority of the interviewed patients had high levels of education, which may have impacted the results, as this patient group may have been more comfortable with the digital toolbox and able to use it more fully. This study is conducted in a Danish context, so the findings might not be applicable in all countries globally. Another limitation is that the interviewers were affiliated with the FP-AF study, which may have introduced response bias, as participants might have responded in ways they thought were more desirable.

### Conclusions

Participation in the FP-AF program enhanced patients’ sense of security, empowerment, and knowledge about AF. This improvement was due largely to a combination of patients’ use of the Heart Portal and the educational sessions at health care centers. Telerehabilitation for patients with AF may be a useful way of researching this group of patients with a focus on rehabilitation. Telerehabilitation for patients with AF may be an effective means of offering rehabilitation to this group in the future.

## Abbreviations

AF: atrial fibrillation
CR: cardiac rehabilitation
CTR: cardiac telerehabilitation
ECG: electrocardiogram
FP-AF: Future Patient—Telerehabilitation of Patients with Atrial Fibrillation
QoL: quality of life
SDT: self-determination theory
